# Supplementary material for: Investigating isoform switching in RHBDF2 and its role in neoplastic growth in breast cancer
Source: PeerJ. 2022 Nov 25;10:e14124. doi: 10.7717/peerj.14124 (PMC9703992; doi:10.7717/peerj.14124)
Supplement: Supplemental Information 6 [file peerj-10-14124-s006.docx]

| **Accession no** | **No. of samples** | **Normal / Tumour** | **Platform** | **Sample** |
| --- | --- | --- | --- | --- |
| **GSE52194** | 19 | 3N / 16T | Illumina Hiseq 2000  (GPL11154) | PT (primary tumour) |
| **GSE130660** | 6 | 3N/3T | Illumina Hiseq 2500  (GPL16791) | PT (primary tumour) |
| **GSE69240** | 35 | 10N / 25T | Illumina Hiseq 2000  (GPL11154) | *DCIS* (Ductal carcinoma insitu) |
| **GSE110114** | 12 | 3N / 9T | Illumina Hiseq 2500 (GPL16791) | IDC (Invasive ductal carcinoma) |
| **GSE45419** | 27 | 3N/ 24T | Genome Analyzer 11x (GPL10999) | IDC (Invasive ductal carcinoma) |
| **GSE51124** | 34 | 10N/24T  (12 G2, 12 G3) | Illumina Hiseq 2000  (GPL11154) | Grade 2 & Grade 3 |
| **GSE148991** | 11 | 5N / 6T | Illumina Hiseq 2500  (GPL16791) | CTCs (circulating tumour cells) |

Table S1: ***The selected RNA-seq datasets and their attributes.***
